# Event and Comment

**Published:** 1910-09-15

**Authors:** 


// DENTAL REGISTER.

Vol. LXIV. September 15, 1910. No. 9.

### EVENT AND COMMENT.

NUMBER OF DENTAL GRADUATES FOR 1909 AND 1910. We print below a list of graduates for the past two years showing some interesting comparisons.

| | 1909 | 1910 |
|---|---|---|
| Harvard University | 10 | 18 |
| Tufts College | 58 | 41 |
| New York College of Dentistry | 61 | 59 |
| New York College of Dental and Oral Surgery | 35 | 29 |
| Buffalo University | 19 | 17 |
| University of Pennsylvania | 114 | 150 |
| Pennsylvania Dental College | 54 | -------- |
| Philadelphia Dental College | 48 | 28 |
| Medico-Chirurgical College | 32 | 49 |
| Pittsburg University | 38 | 24 |
| Baltimore College of Dental Surgery | 52 | 38 |
| University of Maryland | 60 | 54 |
| Baltimore Medical College | 30 | 20 |
| Howard University | 19 | 18 |
| Georgetown University | | 12 |
| Virginia University Dental College | 10 | 3 |
| George Washington University | 7 | 6 |
| Richmond University College of Dentistry | 8 | 11 |
| Atlanta Dental College | 72 | 51 |
| Southern Dental College | 38 | 49 |
| Vanderbilt University | 38 | 31 |
| University of Tennessee | 11 | 10 |
| Maharry Dental College | 19 | 26 |
| Birmingham Dental College | 14 | 11 |
| University of Memphis | | 3 |
| New Orleans Dental College | 18 | 31 |
| University of Southern California | 26 | 15 |
| Texas Dental College | 15 | 15 |
| Texas State Dental College | 5 | 17 |
| Louisville Dental College | 38 | 44 |
| Ohio College of Dental Surgery | 34 | 28 |
| Cincinnati College of Dental Surgery | 8 | 4 |
| Starling Ohio Medical College | 31 | 27 |
| Western Reserve University | 16 | 20 |
| Michigan University | 50 | 48 |

| | 1909 | 1910 |
|---|---|---|
| Indiana Dental College | 49 | 49 |
| Chicago College of Dental Surgery | 84 | 82 |
| Northwestern University | 84 | 87 |
| Illinois University | 29 | 21 |
| Marquette University | 21 | 23 |
| Wisconsin College of Physicians and Surgeons | 9 | 4 |
| University of Minnesota | 50 | 44 |
| Iowa University | 36 | 42 |
| Drake University | 2 | 4 |
| Washington University | 24 | 26 |
| St. Louis College of Dentistry | 18 | 25 |
| Barnes University | 4 | 6 |
| Kansas City Dental College | 28 | 35 |
| Western Dental College | 34 | 42 |
| Lincoln Dental College | 9 | 14 |
| Creighton University | 35 | 19 |
| Colorado College of Dental Surgery | 22 | 20 |
| North Pacific Dental College | 31 | 21 |
| University of California | 15 | 16 |
| San Francisco College of Physicians and Surgeons | 6 | 12 |

| | |
|---|---|
| Total number of schools | 54 |
| Graduates for the year 1909 | 1679 |
| Graduates for the year 1910 | 1599 |

From this it will be seen that the 54 Dental Colleges of the United States have graduated this year 80 less students than were graduated last year. In 1908, 2061 were graduated; in 1905, 2627; the largest number ever graduated in any year.

There has been a gradual shrinkage during the past three years, which does not seem logical in view of the fact that dentistry is continually broadening its sphere of usefulness and activity and that the people are better informed than ever before as to the needs of dentistry, and also that our population has increased each year at the rate of a million people, requiring 200 new dentists, to say nothing of the natural losses from death and retirement. It looks very much as though we are on the eve of an educational revival, as there are not at present dentists enough and with the colleges demanding higher standards we shall necessarily raise the professional standard.

THE NEW NATIONAL ASSOCIATION.—There is probably no subject of more importance to be carefully considered by the profession at the present time than the reorganization of our National Society. It would seem that the art or technical aspect of dentistry had practically reached satisfactory attainments, but the scientific side, while not neglected, does not show desirable activity or progress. Those who have kept closest in touch with professional progress believe the reason for this is to be found in the fact that o substantial appreciation is given to careful literary or scientific work. There is no financial compensation for this sort of effort and probably never will be, and there is no adequate opportunity for the presentation of the results of scientific or literary efforts to any considerable or appreciative audience of the profession. And what is still more deplorable is the fact that no proper stimulus is made by our present literary organizations which will result in the training of men in these times of activity. The organization of the profession into a more systematic and harmonious working body, such for instance, as the Illinois State system, seems to be the most promising effort that has ever been made and from this plan, which is being successfully inaugurated in several other states, has come the idea that our National Association needs to be reorganized to enable it to accomplish the work expected of it in a more satisfactory manner than has heretofore been the case. Many complaints have been made that the present Association is controlled by professional politicians; that it is used to promote commercial interests rather than professional; that its membership is too small and not representative; and other equally ungenerous and untenable theories are offered by those who are disgruntled for some fancied or personal reasons. But after all we could hope and reasonably expect, we should not to day have so united and respected a profession except for the great work done by the Old American and National Dental Associations. The time, however, seems ripe for some change and we must think the subject over in the broadest possible way, and by concentrating the wit and wisdom of all we should evolve some form of organization which shall in large measure represent our present ideals and influence the future of our profession. We desire herewith to contribute our ideas for the reorganization.

A PLAN FOR A NEW NATIONAL ASSOCIATION.—Those who attended the recent meeting at Denver got some idea of what was involved in the way of travel and expense, in time and money for members of the profession living on the Pacific Coast who may have heretofore desired to attend a meeting held in Chicago or Boston; and the fact was made evident that some more convenient and less expensive system was necessary to secure the benefits of a National meeting for the large mass of the profession who could not afford to have much personal interest under the present arrangements. Nine hundred dentists attended this meeting most of them from the near by States. With this condition and so convincing a demonstration we believe the solution of the problem will be found in the formation of four distinct National organizations. One for the east made up of the following States: Maine, New Hampshire, Vermont, Massachusetts, Rhode Island, Connecticut, New York, Pennsylvania, New Jersey, Delaware, Maryland, District of Columbia, Virginia and West Virginia, with Asbury Park as a meeting place. One for the South, constituted of the following States: North Carolina, South Carolina, Tennessee, Georgia, Florida, Alabama, Mississippi, Louisiana, Arkansas, Indian Territory, Oklahoma and Texas, with New Orleans as a meeting place. One for the North, constituted by the following States: Ohio, Kentucky, Indiana, Michigan, Wisconsin, Illinois, Missouri, Iowa, Minnesota, North Dakota, South Dakota, Nebraska, and Kansas, with Chicago as a meeting place. The fourth for the Pacific Coast, constituted from the following States: Montana, Idaho, Wyoming, Colorado, Utah, New Mexico, Arizona, California, Nevada, Oregon, and Washington, with a meeting place at Salt Lake City.

Such an arrangement would provide a meeting within reasonable distance every year for the profession of the entire country, and these meetings by concerted action could be held at the most seasonable time in the year for the several locations, and so as not to conflict with the other district meetings, thus enabling any one so disposed to attend as many of these as he saw fit. For instance the Southern meeting could be held in January, enabling northern men to attend; the Western meeting in April; the Northern in June; and the Eastern in August. We believe that each one of these districts by affiliating all the State and local societies in its territory could have a much larger attendance each year than the present National meeting has ever held. The Eastern society should have a membership of 5000 the Northern 7000, the Southern 3000, the Western 3000 or a combined membership of 18000.

Now these four societies and their component State societies must have some National bond for effective and aggressive promotion of general professional interests. In order that this may be representative and efficient, we suggest under some such title as The National Dental Association Congress, or Congress of the United States Dental Associations, a body made up of one delegate from each State society and three delegates from each of the four District societies, which shall meet annually at some central point, say, St. Louis, and enact such legislation as may from time to time be needed for the best interests of the profession and its several organizations. No literary or technical papers should be read or discussed at such meetings except such as may be necessary to increase the interest in society or business methods of the various organizations connected with the profession. Such matters as educational standards, State Laws, Public Hygiene, Interstate and International Reciprocity, Publication of a Dental Journal, Professional Relations with Commercial and Manufacturing Interests, Patent Rights and Infringements, Army and Navy Affairs, National Library and Museum, Index of Literature, and Encouragement of Scientific Research, etc. In fact this organization should not only serve to unite the whole profession into an effective machine of usefulness, but also serve as a clearing house for all important affairs of National and International concern. It could have charge of all International Congress organizations and work of similar character.

The question of financing these several organizations will be one for the Congress to work out and perfect, but to show its practicability we would suggest that each member be assessed a fee of five dollars each year which would make him a member of his local, state and district societies and entitle him to all privileges in any of them, including the Congress, and also to a copy of the National Journal, in which should be printed all the papers of the District Societies and the best of the State Society papers so far as this might be practicable. The five dollars could be distributed as follows: one dollar to the Local society, two dollars to the State society, one dollar to the District society and one dollar to the Congress. This fee would not be excessive, and every dentist could well afford it, and if anything like the number of dentists we have mentioned above would join the combined societies, ample funds for all needs would be available. We do not know of anything that stimulates men to read and work professionally so much as hearing the discussion of papers and seeing the demonstrations of others in societies. The next best thing is to be able to read of these efforts in the Journal, which ought to be so edited as to make the strongest possible appeal to men who do not attend the meetings, and by a properly conducted journal the doings of the entire profession could be placed in the hands of every member at frequent and regular intervals. It is not necessary for us to comment on the desirability of such a compact and inclusive organization, as it can easily be seen that tremendous undertakings would be possible. We believe also that opportunity for viscious political and personal meddling would be minimized. Let's all offer our suggestions for the convention that is to meet this winter to consider this important matter.

